# Vector Bending Sensor Based on Power-Monitored Tapered Few-Mode Multi-Core Fiber

**DOI:** 10.3390/s26020607

**Published:** 2026-01-16

**Authors:** Qixuan Wu, Zhuyixiao Liu, Hao Wu, Ming Tang

**Affiliations:** Wuhan National Laboratory for Optoelectronics, Optics Valley Laboratory, School of Optical and Electronic Information, Huazhong University of Science and Technology, Wuhan 430074, China; u202111190@hust.edu.cn (Q.W.); d202280963@hust.edu.cn (Z.L.); tangming@hust.edu.cn (M.T.)

**Keywords:** multi-core few-mode fiber, fused taper method, power detection, vector sensing, machine learning

## Abstract

We propose a vector bending sensor based on a tapered few-mode multi-core fiber (FM-MCF). A seven-core six-mode fiber is tapered with an optimized taper ratio, enabling bending sensing through power monitoring. When the tapered FM-MCF bends, coupling occurs between the central core and side cores in the tapered region. By monitoring the power of all cores and employing a power differential method, the bending direction and curvature can be reconstructed. The results show that within a curvature range of 2.5 m^−1^ to 10 m^−1^, the sensitivity of the ratio of the side core’s power to the middle core’s power with respect to curvature is not less than 0.14/m^−1^. A deep fully connected feedforward neural network (DNN) is used to demodulate all power information and predict the bending shape of the optical fiber. The algorithm predicts the bending radius and rotation angle with mean absolute errors less than 0.038 m and 3.087°, respectively. This method is expected to achieve low-cost, high-sensitivity bending measurement applications with vector direction perception, providing an effective solution for scenarios with small curvatures that are challenging to detect using conventional sensing methods.

## 1. Introduction

Small, accurate, and highly sensitive bending sensors play an indispensable role in application scenarios such as collision detection in soft-material robotic systems [1,2,3], kinesiology monitoring in smart wearable devices [4,5,6], health monitoring of bridges and roads [7,8,9], and shape estimation in minimally invasive surgery [10,11]. The advantages of optical fiber bending sensors, such as corrosion resistance, electromagnetic interference resistance, small size, and light weight, make them promising in bending measurement. Traditional fiber bending sensing usually uses lasers to write parallel gratings on the surface of the fiber, such as Bragg gratings [12,13,14], long-period gratings (LPGs) [15], etc. When the fiber is deformed, the grating period and refractive index change, causing wavelength shifts. Various interference structures [16], such as Mach–Zehnder interferometer [17], Fabry–Perot interferometer [18], etc., use phase difference to restore fiber bending. At present, many new bending sensors combine various special optical fibers using the principle of traditional sensors, including twisted helical MCF sensors [19,20], seven-core FBG sensors [21], hole-assisted three-core fiber directional coupling (HATCF) sensors [22], etc. These new sensors can achieve high bending sensitivity and measurement of spatial bending vectors within a specific curvature range.

It is worth noting that although these new sensors have high bending sensitivity within a specific curvature range, they also have some problems. For example, they can only maintain high sensitivity at larger curvatures or can detect a narrow curvature range. Generally, high bending sensitivity sensors can only be used in scenes with severe bending. In more subtle bending scenes, their sensitivity will be greatly reduced. Secondly, the current fiber optic bending sensors basically rely on measuring the wavelength of the output light, which has high requirements on the fiber structure. For example, the fiber grating (FBG) requires precision etching. In practical applications, the packaging, protection and stress coupling technologies of optical fibers are relatively complex, which affects the efficiency of their industrial deployment. For extreme environments (such as strong electromagnetic fields, severe vibrations, high radiation, etc.), although the optical fiber itself has certain advantages, complex optical sensing components such as couplers and demodulators are relatively fragile and are easily damaged by mechanical damage or microbending during installation and wiring. Finally, although the traditional fiber optic sensor itself is cheap, its supporting light source, light detector, spectrum demodulator and other equipment are expensive. The construction cost of the entire system is even higher than that of the traditional electrical sensor system, and its technical maintenance and debugging threshold is also high. In short, traditional fiber optic sensors have defects such as high cost, easy damage, and complex operation, which makes them have great limitations in practical applications.

Due to its unique structure and interferometric properties, tapered multi-core fiber (TMCF)-based sensors have become highly promising candidates for fiber optic sensing in recent years. Unlike traditional single-core fiber sensors, the TMCF structure utilizes strong multi-core mode coupling and interferometric effects, giving it extremely high sensitivity to changes in refractive index, temperature, strain, magnetic field, and curvature. For example, a trench-assisted TMCF refractive index sensor exhibits a sensitivity exceeding 35,000 nm/RIU, one of the highest sensitivities reported to date for fiber optic sensors [23]. Furthermore, multifunctional TMCF structures such as MMF-TMCF-MMF achieve temperature sensitivities exceeding hundreds of pm/°C and linear magnetic field responses [24]. More importantly, the TMCF design can support multi-parameter sensing on a compact and easily fabricated all-fiber platform, and its multi-channel, multi-mode sensing characteristics enable two-dimensional vector sensing. Moreover, it exhibits superior linearity and robustness compared to traditional fiber optic sensors.

This paper proposes a vector bending sensor based on a tapered seven-core six-mode fiber, achieving high-sensitivity bending measurements even at large bending radii. When light is input into the middle core of a tapered few-mode fiber, energy coupling occurs between the middle core and the side core, and the degree of this coupling varies with the taper parameters. When the seven-core six-mode fiber in the tapered region is bent with an appropriate taper parameter, the coupling power of the side core can be conveniently used to characterize the bending degree of the fiber. Since the seven-core six-mode fiber has central symmetry, the direction of spatial bending can be determined by simultaneously monitoring the power of the six symmetrical side cores. Comprehensive simulation analysis shows that this method can achieve a curvature sensitivity greater than 0.14/m^−1^ in the curvature range of 2.5 m^−1^ to 10 m^−1^. At the same time, we design a deep fully connected feed forward neural network (DNN) to demodulate the power information of each core, and predict the fiber bending morphology through machine learning training models. Model evaluation shows that the regression network has a good fit for fiber bending prediction. This work significantly improves the curvature measurement range, ensures high bending sensitivity in small curvature scenarios. Since only the fiber power needs to be monitored, the sensor has a simple structure and low cost, and has great advantages in the field of new sensors.

## 2. Principle

### 2.1. Mode Coupling Theory of Multi-Core Optical Fiber

In optical waveguide theory, various transmission modes exist within an optical waveguide that is uniformly distributed along the axial direction. These modes are orthogonal to each other. When optical waveguides are positioned in close proximity, the modes from each waveguide may interfere or couple with one another [25,26,27]. The closer the spacing between adjacent cores in a multi-core optical fiber, the more pronounced the interference. Multi-core optical fibers are generally classified into two categories based on the distance between the cores: weakly coupled multi-core optical fibers and strongly coupled multi-core optical fibers. Although there is currently no explicit standard for this classification [28], it is commonly accepted that multi-core optical fibers with a core spacing greater than 20 μm are considered weakly coupled, where crosstalk between the cores is minimal. In such fibers, each core can typically be regarded as an independent transmission channel. Conversely, multi-core optical fibers with a core spacing less than 20 μm are classified as strongly coupled, where the close core spacing results in significant light coupling between adjacent cores. This coupling leads to random power transfer between cores during transmission, resulting in widespread interference across the entire fiber.

Random coupling in strongly coupled fibers complicates the quantitative characterization of the coupling degree. In this study, we concentrate on waveguide transmission within weakly coupled optical fibers. When the electromagnetic field distribution post-mode coupling does not significantly differ from the pre-coupling state, perturbation theory can be employed to analyze the transmission characteristics of the coupled waveguide. To simplify the model, we consider a coupled mode theory involving two independent cores in an ideal space. This model facilitates the derivation of expressions for mode coupling equations and lateral coupling coefficients under weak coupling conditions. When examining multi-core optical fibers, since coupling primarily manifests in the transverse direction, the transverse mode coupling theory of optical waveguides is typically used to analyze the mode coupling of adjacent cores within the multi-core optical fiber structure [29,30]. Based on the concept of transverse mode coupling, under weak core coupling conditions—where the distance between cores is relatively large compared to the core radius—the cores in the multi-core optical fiber will experience a significant change in the amplitude of the received signal due to coupling by the light field energy from adjacent cores, while the propagation mode within the core remains unchanged. Under weak coupling conditions, the multi-core optical fiber adheres to the mode coupling equation group presented in the following formula.

(1) $$\left\{\begin{array}{c} \frac{da_{1}\left(z\right)}{dz}=j\beta_{1}a_{1}\left(z\right)+jK_{21}a_{2}\left(z\right)\\ \frac{da_{2}\left(z\right)}{dz}=jK_{12}a_{1}\left(z\right)+j\beta_{2}a_{2}\left(z\right) \end{array}\right.$$

(2) $$\alpha_{i}\left(z\right)=c_{i}\exp\left(j\beta_{i}z\right),i=1,2$$

Among them, *a_i_*(*z*) represents the wave terms of the two fiber modes, *c_i_*(*z*) is the slowly varying envelope of the corresponding mode, exp(*jβ_i_z*) is the rapidly varying part of the corresponding mode, and *K_ij_* is the transverse coupling coefficient of each core. When only two adjacent cores in a multi-core fiber are considered, it is abstracted into a dual-core fiber model with only two parallel optical waveguides, core 1 and core 2. Assuming that only core 1 sends an optical signal, its optical field satisfies the following set of equations in the multi-core fiber region.

(3) $$\left\{\begin{array}{l} E\left(r\right)=e_{1}\left(r\right)\exp\left(j\beta_{1}z\right)\\ H\left(r\right)=h_{1}\left(r\right)\exp\left(j\beta_{1}z\right) \end{array}\;\right.$$

Among them, *e*_1_ and *h*_1_ represent the mode field in core 1. Symmetrically, assuming that only core 2 sends optical signals, its optical field satisfies the following set of equations in the multi-core fiber region.

(4) $$\left\{\begin{array}{l} E\left(r\right)=e_{2}\left(r\right)\exp\left(j\beta_{2}z\right)\\ H\left(r\right)=h_{2}\left(r\right)\exp\left(j\beta_{2}z\right) \end{array}\;\right.$$

Among them, *e*_2_ and *h*_2_ represent the mode field in core 2. According to the approximate weak coupling condition, under the theoretical assumption, the area where the two parallel cores in space are located can be described as the electromagnetic field of the composite mode composed of two optical waveguides, and its distribution is expressed as shown in the following formula.

(5) $$\left\{\begin{array}{c} E_{m}=c_{1}e_{1}\exp\left(j\beta_{1}z\right)+c_{2}e_{2}\exp\left(j\beta_{2}z\right)\\ H_{m}=c_{1}h_{1}\exp\left(j\beta_{1}z\right)+c_{2}h_{2}\exp\left(j\beta_{2}z\right) \end{array}\right.$$

Among them, *c*_i_ will change with *z*, but it is assumed here that it changes slowly. On this basis, the expression of Em is derived from Maxwell’s equations and expanded in core 1 and core 2; and the cladding part outside the two cores. Since energy is conserved in the ideal dual-core fiber model, there is no loss in the transmission of light in the waveguide, and mode coupling only occurs between the two cores 1 and 2 in the optical fiber, no matter how the two cores are coupled, the total power in the two cores remains unchanged. At the same time, since the refractive index parameters of core 1 and core 2 are exactly the same, *K*_12_ = *K*_21_, and the mode coupling equation can be simplified as Formula (6)

(6) $$\left[\begin{array}{c} a_{1}\left(z\right)\\ a_{2}\left(z\right) \end{array}\right]=e^{j\beta z}\left[\begin{array}{cc} \cos\left(\mathrm{kz}\right) & \mathrm{jsin}\left(\mathrm{kz}\right)\\ \mathrm{jsin}\left(\mathrm{kz}\right) & \cos\left(\mathrm{kz}\right) \end{array}\right].\left[\begin{array}{c} a_{1}\left(0\right)\\ a_{2}\left(0\right) \end{array}\right]$$

The mode coupling coefficient, denoted as *k*, is solely dependent on the lateral distribution of the mode field and is independent of the longitudinal component *z*. The energy within fiber cores 1 and 2 alternates periodically between the two modes, akin to two parallel optical waveguides. In other words, under ideal conditions, the energy in the two fiber cores will not attain a stable state during transmission but will exhibit periodic fluctuations. This contrasts with the actual conditions of optical fiber transmission. The primary reason for this discrepancy is that, in practice, light energy diminishes as it travels further along the waveguide. Additionally, mode coupling is not confined to the same mode. Consequently, in the real-world optical waveguide transmission process, after light energy has traversed a sufficiently long waveguide, mode coupling will eventually settle into a stable state.

### 2.2. Mode Coupling During Bending

When a multi-core fiber (MCF) is bent, optical energy is coupled from one core to another due to the change in the mode within the fiber. The specific coupling mechanism and coupling efficiency depend on the fiber’s geometry, bending radius, spacing between cores, and the operating wavelength of the fiber. Next, we discuss the mathematical model of optical energy coupling caused by bending.

Idealistically, each core of a multi-core fiber supports distinct light modes, and these modes exhibit specific propagation characteristics when transmitted through the fiber. When the fiber is bent, the direction of light propagation changes, the refractive index within the fiber alters, and the light field also changes, resulting in mode coupling between the fiber cores. Among the factors affecting the degree of mode coupling are the inter-core distance (*d*), the bending radius (*r*), the core size and numerical aperture (NA) of the fiber, and the wavelength (*λ*). The following formula is typically used to estimate the coupling efficiency between cores, which is the efficiency of coupling light energy from one core to another.

(7) $$\eta_{ij}\left(z\right)={\left|\sin\left(\kappa_{ij}z\right)\right|}^{2}$$

*η*_ij(z)_ is the coupling efficiency from core *i* to core *j*. *κ*_ij_ is the coupling coefficient between core *i* and core *j*. z is the propagation distance. During mode coupling, the change in mode amplitude *a*_i_(*z*) satisfies the coupled wave equation:

(8) $$\frac{da_{i}\left(z\right)}{dz}=\underset{j\neq i}{\sum }i\kappa_{ij}a_{j}\left(z\right)e^{i\Delta \beta_{ij}z}$$

*a_i_*(*z*) is the mode amplitude of the *i* th core; $\kappa_{ij}$ is the coupling coefficient between cores *i* and *j*; $\Delta \beta_{ij}$ = β*_i_*− β*_j_* is the mode phase mismatch between the two cores; *z* is the propagation distance. The expression of the coupling coefficient $\kappa_{ij}$ is [31]

(9) $$\kappa_{ij}=\frac{\omega }{2}\iint \Delta n\left(r\right)\psi_{i}^{*}\left(r\right)\psi_{j}\left(r\right)d$$

Δ*n*_(*r*)_ is the refractive index perturbation caused by bending; $\psi_{i}^{*}\left(r\right)$ and $\psi_{j}\left(r\right)$ are the mode field distributions; ω is the angular frequency. In commonly used fundamental mode equivalent refractive index models, a bend with radius R can be represented as a transverse index variation along the bending direction x, $n_{\mathrm{eq}}\left(x\right)\approx n\left(x\right)\left(1+x/R\right)$, yielding $\Delta n_{\mathrm{bend}}\left(x;R\right)\approx n\left(x\right)x/R=n\left(x\right)\;x\;\kappa$, with κ=1/R. Bending also introduces stress that can be linked to refractive-index change through the photo elastic effect. In this work, the bending-induced index variation is implicitly accounted for by the BeamPROP bending-waveguide solver, and the resulting inter-core power redistribution is used for sensitivity evaluation and dataset generation.

The impact of the bending radius r on mode coupling is evident through the coupling coefficient and phase mismatch. From the viewpoint of the coupling coefficient, bending decreases the spatial separation between the mode fields of adjacent cores, thereby increasing their overlap. As for phase mismatch, bending modifies the effective refractive index of the modes, which in turn influences the phase difference between them. The exact values of these parameters are contingent upon the fiber structure and bending conditions, necessitating numerical simulations or detailed calculations for precise determination.

## 3. Math Simulation of Tapered Seven-Core Six-Mode Optical Fiber Based on Rsoft

### 3.1. Model Establishment

**Figure 1:**
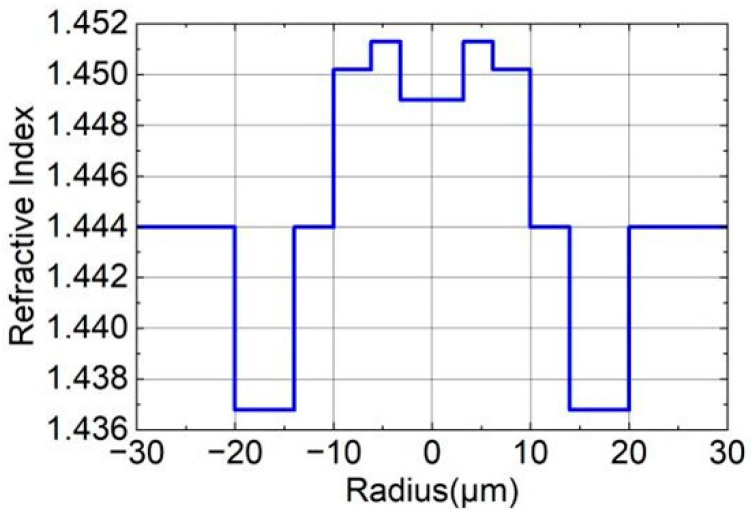
Radial distribution of refractive index of seven-core six-mode optical fiber.

The seven-core six-mode optical fiber used in this paper has a core spacing of 42.5 μm and a cladding diameter of 150 μm. Each core is a six-mode optical fiber with a specially designed multi-cladding structure. Figure 1 gives the refractive index distribution of a single core and the specific parameter values. This design enables the optical fiber to have a high degree of isolation.

**Figure 2:**
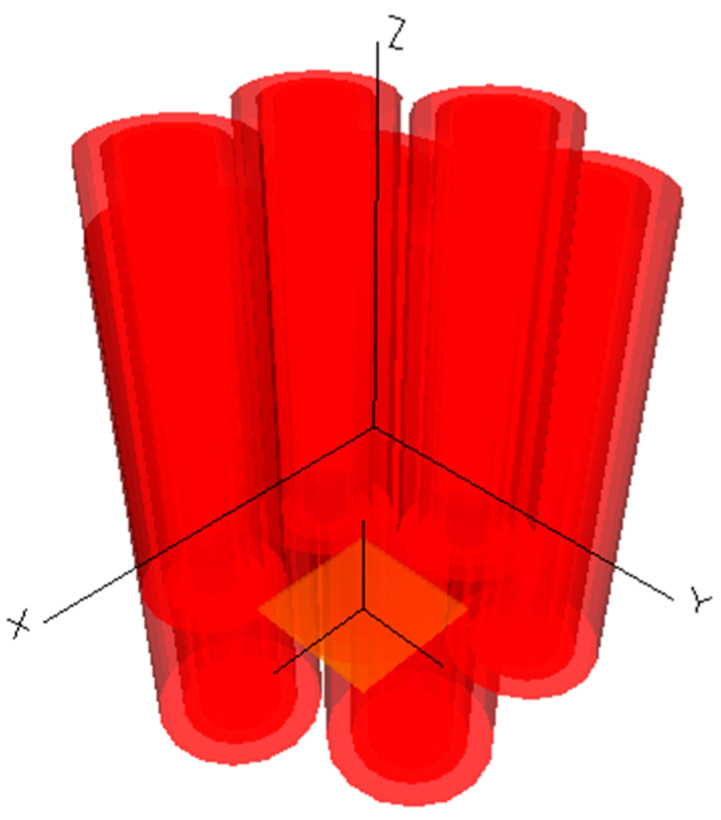
3D model of seven-core six-mode optical fiber.

Considering that mode coupling occurs between the cores of a multi-core optical fiber, the energy transfer between cores is related to the cross-sectional structure and transmission distance of the multi-core optical fiber. Therefore, we choose to establish a three-dimensional optical waveguide model. As shown in Figure 2, the three-dimensional modeling is performed through the BeamPROP module of the RSOFT simulation software (RSoft Photonics CAD Suite Version 2018.12, 64-bit). The BeamPROP module utilizes the Finite-Difference Beam Propagation Method (FD-BPM) to simulate and analyze optical devices. It is specifically used to design integrated optical waveguide components and optical paths. It is a professional module that highly integrates computer-aided design and simulation.

After the geometric model is established, it is necessary to define the parameters required for the simulation. These include the size of the geometric model, coordinates, cross-sectional refractive index distribution, and working wavelength. Based on the refractive index distribution of the optical fiber core end face shown in Figure 1, the distribution of the cross-sectional refractive index of the component model is defined as a step type. This means the refractive index changes abruptly at the core-cladding interface. Then, other variables are set. The ratio of the core diameter before tapering to the core diameter after tapering is defined as *N*, referred to as the taper ratio, and its value will be discussed later. The core spacing *A* after taper is set to 42.5/*N* μm, the core diameter is 19.9 × 2/*N* μm, and the core length L is 10 mm. Additionally, the step refractive index distribution is divided into five layers *A*_1_*–A*_5_. The diameters of *A*_1_*–A*_5_ are 3.188 × 2/*N* μm, 6.257 × 2/*N* μm, 9.9 × 2/*N* μm, 13.9 × 2/*N* μm, and 19.9 × 2/*N* μm, respectively. The refractive index differences (including the cladding refractive index) Δ_1_*–*Δ_5_ are set to 0.005, 0.0074, 0.0062, 0, and 0.0072. The working wavelength is set to 1.55 μm, the background refractive index (LP_01_) to 1.444, and the effective refractive index (LP_01_) of the fiber without tapering to 1.44964.

Note that although the optical field is excited in the fundamental LP01 mode at the fiber input under defined and repeatable excitation conditions, this does not mean that the sensing process occurs in a single-mode state. Due to perturbations caused by the tapered structure and bending, the effective refractive index of the guided modes decreases along the propagation direction, leading to significant inter-core coupling and inter-mode mixing within the tapered region. Therefore, higher-order modal components (e.g., LP11 and LP21 types) can be naturally excited and participate in power redistribution during propagation.

It is important to emphasize that the proposed sensing scheme is based on monitoring the total output power of each core, rather than resolving individual modal components. Therefore, the measured core power represents the combined contribution of all guided modes supported by that core. Thus, the influence of higher-order modes is implicitly included in the extracted power features and subsequent neural network demodulation without explicit mode-by-mode separation or analysis. In strongly coupled conical regions, the evolution of the optical field is better described by coupled multi-core states under bending perturbation, and the resulting redistribution of intercore power constitutes the physical basis of the proposed vector bending sensing method.

### 3.2. Optimization of Fused Taper Ratio and Taper Length

In this simulation design, the core spacing of the seven-core six-mode fiber is 42 μm, which belongs to a weakly coupled multi-core fiber. In the absence of external interference, energy coupling between the cores does not occur, or it is considered that the energy coupling is very weak and can be ignored. Therefore, to control the energy coupling in the FM-MCF, we propose reducing the core spacing using the fused taper method (fiber taper) to promote energy coupling, thereby facilitating the transfer of energy from the middle core to the side cores. By adjusting the ratio of the fiber core diameter after tapering to the diameter before tapering, known as the tapering ratio, the power distribution of each fiber core can be tailored to achieve optimal bending sensitivity. The fused taper method involves combining two (or more) uncoated optical fibers in a specific manner, melting them under high temperature, and stretching them simultaneously to both sides, ultimately forming a special double-tapered waveguide structure in the heating area. Different splitting ratios can be achieved by controlling the twist angle and stretching length of the optical fiber.

**Figure 3:**
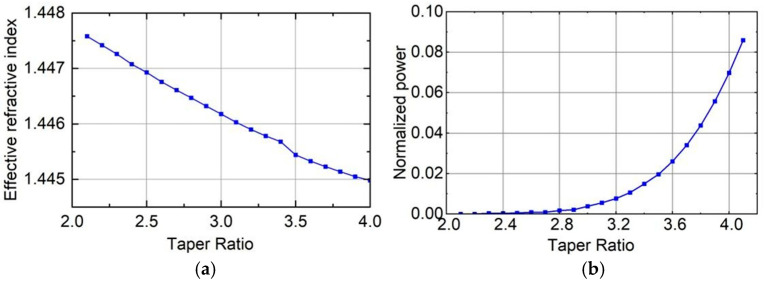
(**a**) The trend of effective refractive index changing with taper ratio; (**b**) The change in FM-MCF side core power when *z* = 0.

In order to promote the energy coupling phenomenon in the FM-MCF within the allowable range of mechanical strength, we strive to control the core spacing to 20 μm or less, which requires the taper ratio (*N*) to be at least 2.1 (42.5/20). Then, we increase the taper ratio by 0.1 each time and use the mode analysis module to determine the mode field of the fundamental mode during single-core transmission, as shown in Figure 3. The horizontal axis represents the taper ratio, which increases from 2.1 to 4.0 in increments of 0.1; the vertical axis represents the effective refractive index of the fundamental mode (LP_01_) in the fiber core. As shown in the figure, the effective refractive index of the fiber decreases as the taper ratio increases.

When the effective refractive index of the mode within the optical waveguide approaches that of the cladding, the stability of the core-cladding structure is compromised, leading to light being predominantly transmitted through the cladding. However, due to significant attenuation within the cladding, stable transmission cannot be sustained in the optical fiber. In a stable fundamental mode transmission state, light is injected into the central core, and theoretically, the power of the side core at the transmission distance *z* = 0 within the core should be nearly 0. Nevertheless, if the effective refractive index of the transmission mode is excessively low and nearly matches that of the cladding, the power of the side core at *z* = 0 will not be negligible. When the input optical mode field energy is fixed at 1, and the taper ratio increases incrementally from 2.1 to 4.0, the variation in the side core power of the FM-MCF at *z* = 0 is depicted in Figure 3. As illustrated, when the taper ratio surpasses 2.8, the power of the side core at *z* = 0 begins to rise notably, suggesting that when the taper ratio exceeds 2.8, the fundamental mode within the core of the seven-core six-mode optical fiber can no longer remain stable and starts to transition into the cladding mode. Consequently, the maximum taper ratio for subsequent simulations is set to 2.8.

**Figure 4:**
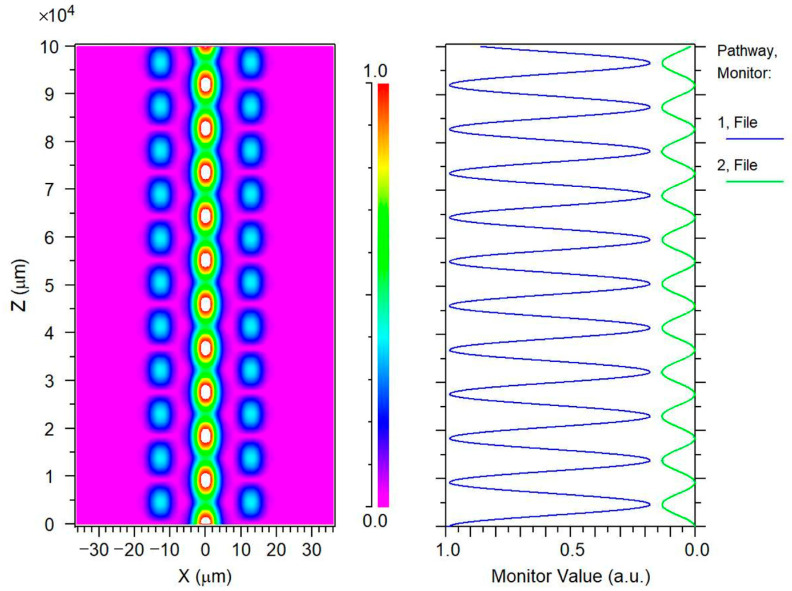
Power variation when the middle and side cores are not bent (taper ratio is 2.8).

To observe the energy transition between the central core and the peripheral cores during the transmission of input light through a tapered FM-MCF, we monitor the power levels of both the central core and the peripheral cores. When the fiber remains unbent, the six peripheral cores of the seven-core six-mode fiber exhibit central symmetry and are equivalent in function, thus necessitating the monitoring of only one peripheral core. Consequently, we focus on the power of the central core and one peripheral core in the tapered region with a taper ratio of 2.2, as illustrated in Figure 4.

**Figure 5:**
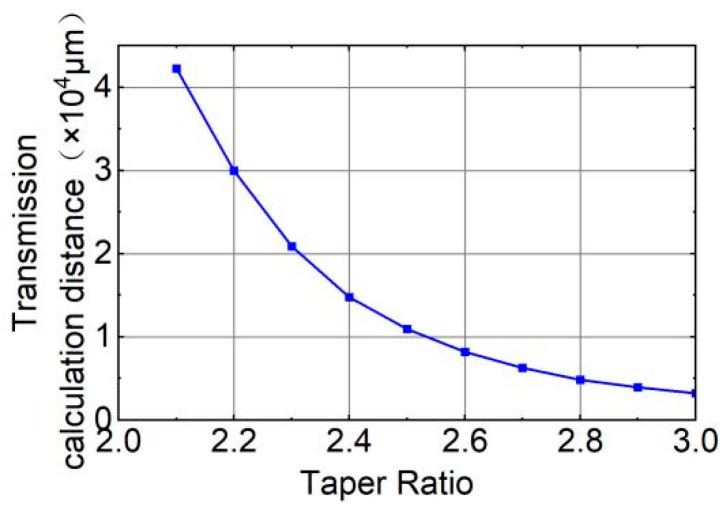
The variation trend of transmission calculation distance with taper ratio.

As the energy of the middle core diminishes, the energy of the side core intensifies. This occurs when the power from the middle core dissipates towards the side core and the cladding, causing the side core’s power to gradually rise. When the power of the middle core hits its lowest point, the side core’s power simultaneously peaks. Under ideal conditions, without accounting for any losses, the energies of the middle and side cores will not stabilize during transmission but will exhibit periodic fluctuations, aligning with theoretical predictions. We refer to the juncture where the power of both the middle and side cores reaches its extreme values simultaneously as the “coupling point.” The position of this “coupling point” along the optical fiber’s radial direction correlates with the taper ratio. As depicted in Figure 5, the horizontal axis represents the taper ratio, while the vertical axis indicates the transmission calculation distance from the optical fiber’s input end (*z* = 0) to the first “coupling point.” Here, the “coupling point” is defined as the propagation location where the power of the central core reaches its first minimum, while the power of the adjacent side cores simultaneously reaches its maximum. The power coupling effect between the cores is strongest at this location. With an increasing taper ratio, the distance required to reach the “coupling point” decreases. This implies that as the taper ratio increases, the smaller the core spacing in the seven-core six-mode optical fiber, the more likely mode coupling is to occur, and the higher the degree of coupling. This observation is consistent with the theoretical principles outlined earlier. In practical experimental settings, the taper length typically does not surpass 8 cm. Within the taper ratio range of 2.2–3.0, complete coupling can happen at least once within the fiber’s taper region.

Based on the above analysis, to improve the coupling efficiency and coupling degree, and ensure that the optical fiber has higher bending sensitivity, a larger taper ratio should be used during the stretching process. However, as shown in Figure 3, when the taper ratio exceeds 2.8, the fundamental mode in the core of the seven-core six-mode optical fiber will no longer be stable. Therefore, we choose 2.8 as the optimal taper ratio. We calculated the transmission distance when the power coupling between the middle core and the side core reaches the maximum value under different taper ratios. The simulation results show that when the taper ratio is 2.8, the core power reaches the “coupling point” after transmitting 4.84 mm. At this time, the coupling degree of the core reaches a peak value and the coupling efficiency reaches a maximum value. In the simulation, we need to measure the power of each core at the end of the fiber taper area. Therefore, the taper length of the seven-core six-mode optical fiber is determined to be 4.84 mm to ensure the highest core coupling degree and bending sensitivity. In summary, the taper ratio (*N*) is set to 2.8 and the taper length is set to 4.84 mm.

## 4. Bending Test

**Figure 6:**
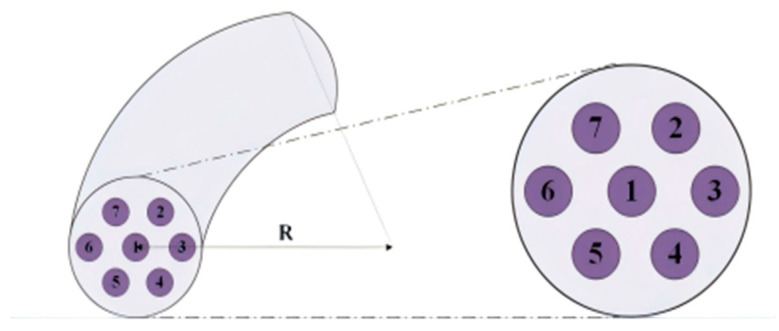
Fiber numbering and bending diagram.

**Figure 7:**
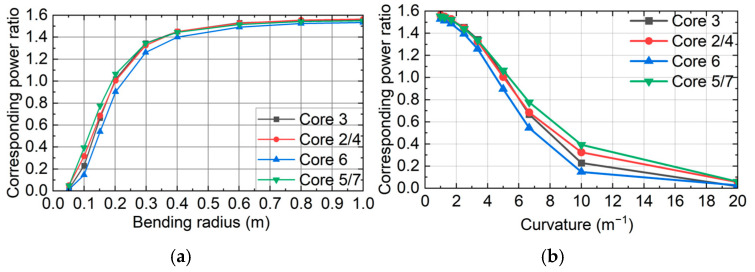
(**a**) Corresponding power ratio changes with bending radius; (**b**) Corresponding power ratio changes with curvature.

Under conditions of a taper ratio of 2.8 and a taper length of 4.84 mm, as depicted in Figure 6, the seven-core six-mode optical fiber is bent in the positive direction (to the right of core No. 3). Considering the optical fiber’s mechanical strength and the actual required bending sensing range, the bending radius is set between 0.05 m and 1 m. The output power of the six side cores is collected at the end of the optical fiber taper. To better reflect the power coupling degree of each side core, we examined the variation in the ratio of each side core power to the middle core power (corresponding power ratio) with the bending radius. To further examine the relationship between the variation in power ratio and the degree of bending, the bending radius is converted into curvature, thereby establishing the relationship between these two parameters. This is depicted in Figure 7.

As the bending radius of the optical fiber varies, the power ratio between the side core and the middle core of the FM-MCF undergoes significant changes. When the bending radius increases, the power ratio of each side core rapidly rises from zero and then gradually stabilizes. This occurs because at an extremely small bending radius (less than 0.05 m), the degree of bending is high, leading to bending loss and other factors causing core power to overflow the cladding. Consequently, the side core power becomes negligible, making it challenging to observe changes in the ratio. Conversely, at a larger bending radius, the degree of bending is reduced, and power coupling approaches the unbent state, thus stabilizing. In the region of moderate bending, the coupling degree of each side core changes rapidly with the alteration of the optical fiber’s bending degree. Along the bending axis, the power ratio of core No. 6, which is closest to the outside of the bend, is consistently the smallest among all the optical fiber cores. Meanwhile, the power ratios of the remaining five side cores are generally similar. Specifically, the power ratios of cores No. 5 and No. 7 are the highest, followed by cores No. 2 and No. 4, with core No. 3 having the smallest ratio.

The curvature is not completely linear when it changes from 1 m^−1^ to 20 m^−1^ and can be divided into three different linear regions: 1~2.5 m^−1^, 2.5~10 m^−1^, and 10~20 m^−1^. The sensitivity of the ratio of the inner core power to the middle core power of different fiber cores in the three regions to the curvature is calculated, as shown in Table 1.

In the three regions, the power ratio of the six side cores within the 1~2.5 m^−1^ and 10~20 m^−1^ ranges is less sensitive to curvature changes. However, the curvature is more sensitive in the 2.5~10 m^−1^ range, making it the optimal bending radius sensing measurement range, which is 0.1~0.4 m. Furthermore, within this range, the sensitivity of core No. 3 and core No. 6 to curvature along the bending axis is higher than that of the other four cores. This occurs because core No. 3 and core No. 6 are positioned on the inside and outside of the bending direction, respectively. When the fiber bends positively, all cores contract along the bending direction, causing cores No. 3 and No. 6 to move closer to the middle core than the other four side cores. Additionally, since core No. 3 has a smaller relative bending radius, its contraction distance along the bending direction is also smaller, resulting in the closest proximity to the middle core. Consequently, core No. 3 is the most sensitive to curvature changes, aligning with coupling theory. From this, we deduce that when an optical fiber bends, the core on the inside of the bending direction is most sensitive to curvature, followed by the core on the outside of the bending direction, then a group of cores with the bending direction as the axis of symmetry and closer to the inside, and finally, the group of cores with the bending direction as the axis of symmetry and closer to the outside, which is the least sensitive to curvature. Based on the central symmetry of the six side cores, the outside of any side core is chosen as the positive bending direction, and the sensitivity of each side core power ratio to curvature aligns with the aforementioned conclusion.

Based on the above data analysis, the bend sensitivity of the tapered seven-core six-mode fiber is greater than 0.14/m^−1^ within the curvature range of 2.5 m^−1^ to 10 m^−1^, with the sensitivity of both cores along the bend direction exceeding 0.16/m^−1^. Compared to traditional bend sensors, the seven-core six-mode fiber achieves highly sensitive bend sensing within the curvature range of 2.5 m^−1^ to 10 m^−1^. Furthermore, by leveraging the medial symmetry of the seven-core six-mode fiber, the bend direction at a fixed curvature can be determined by comprehensively analyzing the power of the cores on each side, thereby achieving vector sensing. The following is a simulation analysis of bend direction sensing.

**Figure 8:**
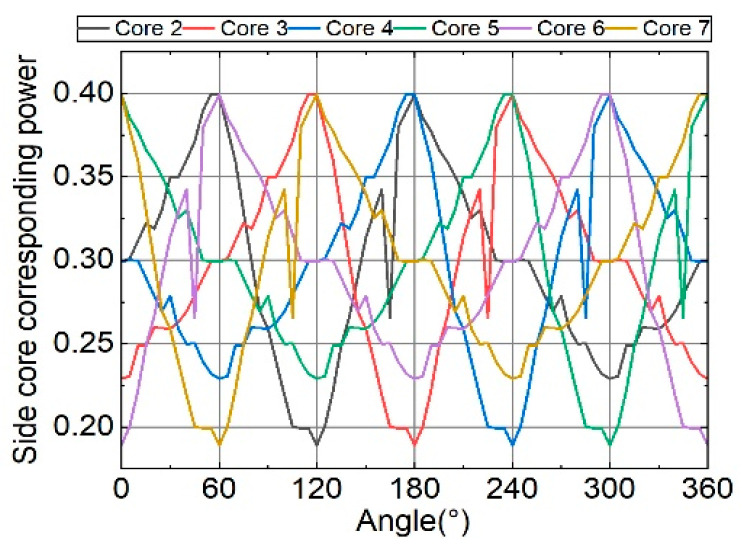
When the input power is 1 and the bending curvature is 10 m^−1^, the change in the side core corresponding power relative to the bending direction.

A rectangular coordinate system is established with the line passing through cores 3 and 6 as the X-axis and the right side of core 3 as the positive direction. The spatial bending vector rotates counterclockwise about the X-axis, and the angle between this vector and the positive X-axis is defined as *α*. When the input power is set to 1 and the curvature is 10 m^−1^, the relationship between the equivalent corresponding power of the six side cores and the rotation angle is studied as the value of α varies from 0° to 360°, as shown in Figure 8. Due to the seven-core six-mode fiber is centrally symmetric, the power in each side core varies with a 60° period. It can be seen that changes in the bend direction cause significant periodic fluctuations in the power of the side cores, which is consistent with the spatial symmetry of the cores in the FM-MCF. By comprehensively analyzing the power variations in the six cores, the power of each side core can be matched to the bend direction in turn, thereby determining the bend direction and achieving perception of the spatial bend vector.

To more clearly and intuitively demonstrate the performance of the proposed sensor, we directly compare its performance with that of mainstream vector bending sensors across five dimensions: sensitivity, measurement range, vector sensing capability, system cost, and structural complexity. As shown in Table 2, the power-sensing-based seven-core six-mode vector bending sensor maintains high sensing sensitivity while possessing a larger curvature sensing range, better vector sensing characteristics, a simpler system structure, and lower system losses. This fully demonstrates the advantages of the proposed sensor.

However, in actual calculations, we found that the factors affecting the side core power variation (core spacing and axial strain) do not change monotonically during rotation. The core spacing and axial strain have different effects on the core power magnitude, and the core power response sensitivity is not uniform at different bend radii. Although the power variation in a single core exhibits clear periodicity, the variation in the core output power with rotation angle within the period is highly complex. This complexity makes traditional function fitting methods inadequate for accurately fitting the relationship between power and bend shape. Therefore, machine learning is introduced to deconstruct the complex power variations and predict the fiber’s bend shape.

## 5. Bending Estimation

To address the challenge of complex fiber core power under varying bending directions and to quantitatively analyze the fiber’s response under different bending conditions, this paper proposes a deep regression framework, BendNet, which aims to reconstruct the bending radius and rotation angle based on measured power values from six side cores. To train BendNet, we constructed a systematic bending database based on the RSoft simulation system.

In each simulation, a seven-core six-mode fiber is bent under given bending curvature and rotation angle, and the output power of the six side cores is recorded at the tapered end. The curvature range in the simulation system is set to 1–20 m^−1^, with 2.5–10 m^−1^ representing the sensor’s high-sensitivity operating range. Curvature is discretely sampled with a fixed step size; simultaneously, the bending direction is rotated and sampled in 1° steps within the range of 0–360°. For each set (curvature, angle), the power of the six side cores is extracted and normalized to min–max, forming the network input; the corresponding bending radius and rotation angle serve as supervision labels. This ultimately forms a training sample set covering the entire working space for the regression training of the neural network. BendNet takes six normalized power values as input and outputs two continuous variables: bending radius and angle. A multi-layer neural network model is used to enhance BendNet’s ability to generalize and fit feature data. This study employs a deep fully connected feedforward neural network (DNN) for batch processing of the data. DNN is a simple yet powerful neural network model widely used in tasks such as regression, classification, and feature modeling.

**Figure 9:**
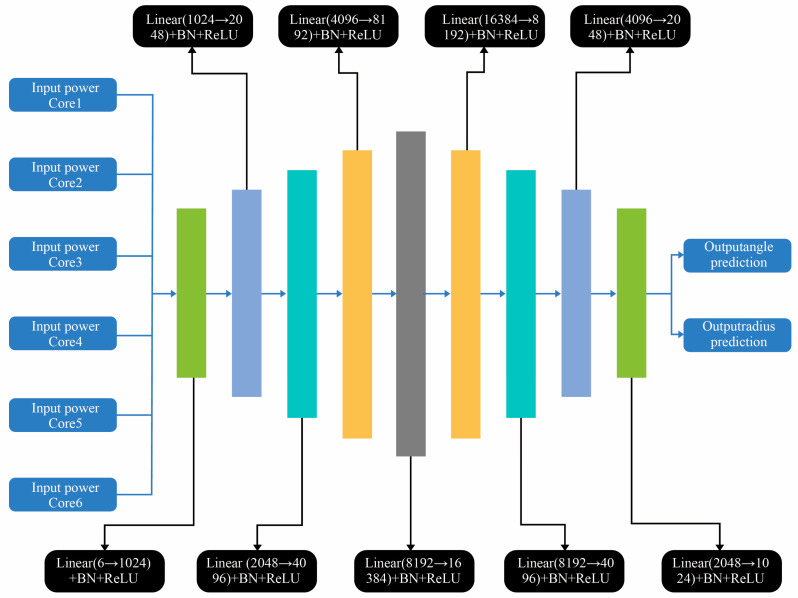
Schematic diagram of the DNN structure used by BendNet.

We use a relatively simple network structure to process the input data, collect the side core output power under different bending radii and rotation angles, extract the power features (6 dimensions) of each sample and its corresponding label (radius + angle). The power feature is normalized by the min-max of the sample. And the radius and angle in the label are normalized by the maximum value to improve training stability. A symmetric DNN is constructed, and its structure is shown in Figure 9. It starts with a 6D input layer, expands to a central hidden layer with up to 16,384 neurons, and then compresses to a 2D output layer. Batch normalization (BatchNorm) and ReLU activation are performed after each layer to prevent gradient vanishing, overfitting, and other problems, promote convergence, and alleviate internal covariate shift. This high-capacity design enables the network to capture the subtle nonlinear mapping between power distribution and spatial curvature features.

**Figure 10:**
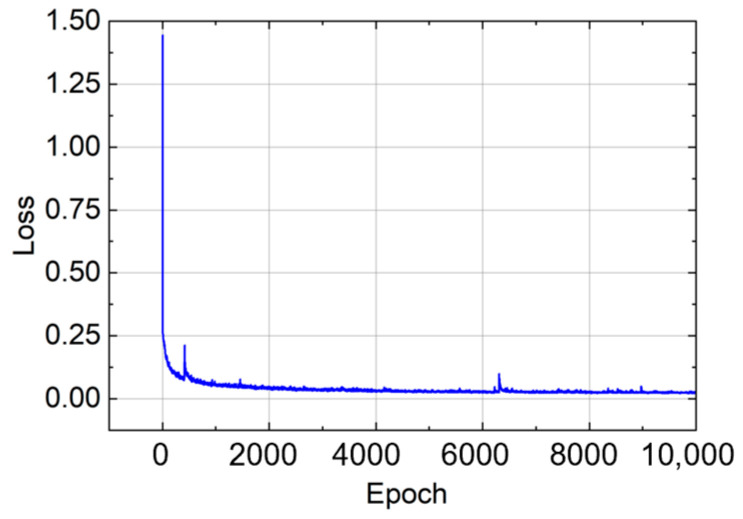
BendNet training loss.

The model was trained using the Adam optimizer and L1 loss function, with a training set of 10,000 epochs and a batch size of 2520. The model with the smallest validation loss was saved and selected for evaluation, and the model training loss is shown in Figure 10. After training, the predicted values were denormalized using the scale factor obtained during preprocessing. The model performance was evaluated using mean absolute error (MAE), root mean square error (RMSE), and coefficient of determination (*R*^2^). For bend radius prediction, the model achieved a MAE of 0.038 m, a RMSE of 0.084 m, and an *R*^2^ of 0.9275. For rotation angle prediction, the MAE was 3.087°, the RMSE was 20.263°, and the *R*^2^ was 0.9581.

**Figure 11:**
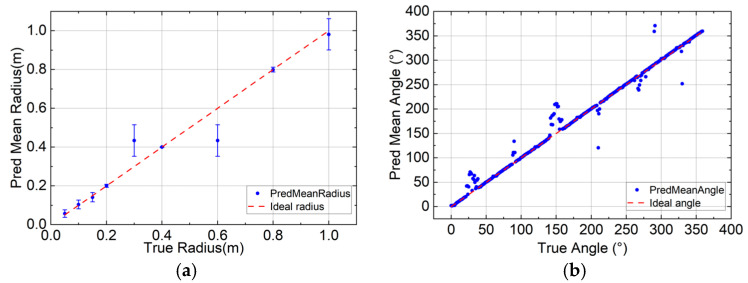
(**a**) Bending radius error bar graph; (**b**) Rotation angle prediction mean graph.

**Figure 12:**
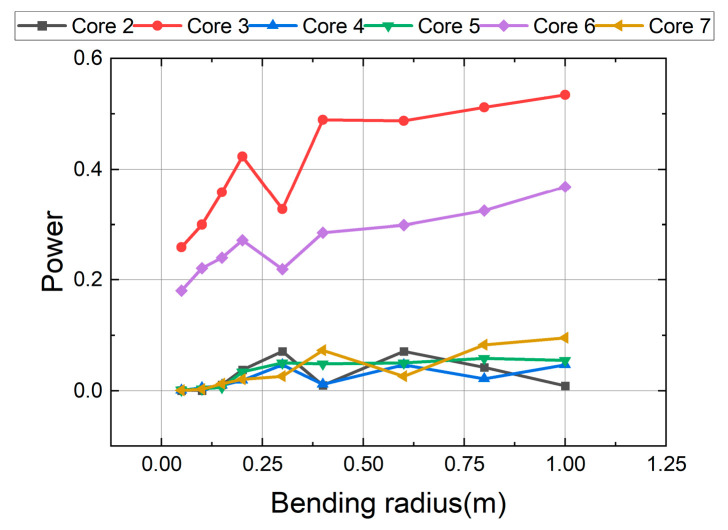
Distribution of power of each core as a function of bending radius when the rotation angle is 45°.

To more intuitively demonstrate the model’s predictive performance, we plotted an error bar chart for the bending radius and a prediction mean chart for the rotation angle, as shown in Figure 11. The figures display the prediction mean and standard deviation to aid in understanding the model. It’s important to note that the model exhibits significant errors in predicting the bending radius at radii of 0.3 m and 0.6 m. To explain this, we plotted the power distribution of each core as a function of the bending radius at a rotation angle of 45°. As shown in Figure 12, at 0.3 m, abnormal fluctuations in core power occur. As the bending radius decreases, the fiber curvature increases significantly, leading to enhanced mode coupling and changes in loss. Simultaneously, the axial strain of the fiber core caused by deformation may affect mode coupling. These changes result in significant fluctuations in the power distribution across different cores when the curvature increases significantly. These fluctuations and couplings are random, making model learning relatively difficult. Therefore, the error is larger at this point. The abnormal fluctuations may also be due to the sensor’s response to curvature; this sensor is highly sensitive at small bending radii. This is a characteristic of the simulation model; at such a small bending radius, the measured power changes more drastically, leading to these outliers. At 0.6 m, we observed that the sensor’s bending sensitivity began to decrease, the power response became more stable, and its dependence on small changes in the bending radius decreased. Therefore, the sensor response was less prone to significant changes at this point, and the slope of the power-radius curve decreased. This reduced power sensitivity to bending deformation may lead to a “many-to-one” situation in model learning, thereby reducing the model’s predictive ability and introducing anomalous biases. We attempted to retrain the model by optimizing the sample size, increasing the number of model layers, decreasing or increasing the learning rate, and changing the optimization function, but the results did not show significant improvement. For the reasons mentioned above, increasing the sample size at these two points did not significantly improve the model’s performance. These biases, inherent in bent fiber optic sensing systems, cannot be effectively eliminated by optimizing sampling or training the model.

In terms of the prediction error of the rotation angle, the predicted mean value is offset when rotating 30°, 90°, 150°, 210°, 270°, and 330°. Combined with the spatial intensity distribution analysis of Figure 8, the power of the axisymmetric core is consistent at these angles, and the disappearance of the power difference may cause the model to have errors in judging the bending direction. To further illustrate this, we removed these axisymmetric rotation angles and re-evaluated the model. In rotation angle prediction, the model achieved a MAE of 0.960°, an RMSE of 1.960°, and an *R*^2^ of 0.9998. This also indicates that the model training fit was good, and the high RMSE value mentioned above was significantly affected by a few outliers. In summary, the model has high prediction accuracy for both the bending radius and the rotation angle. The error bar graph and the mean graph of the prediction results both show a nearly ideal linear correlation. The model has a small MAE for the prediction of the bending radius and the rotation angle, and the *R*^2^ is close to 1, which fully demonstrates that the model has a high accuracy in predicting the bending morphology of the optical fiber.

It is worth noting that the model effectively captures the spatial symmetry inherent in the sensor layout. Model evaluation results show that the predicted output remains robust in different bending directions. The algorithmic framework complements the physical sensor design: the tapered fiber structure ensures high sensitivity to power changes caused by bending, and the unique central symmetric structure of the seven-core six-mode fiber enables the sensor to determine the bending direction based on the output power changes in the side cores, while BendNet can accurately and in real time map these changes to the bending shape of the fiber, thereby realizing seven-core six-mode fiber space vector bending sensing.

## 6. Conclusions

To develop a bending sensor that is more direct, efficient and practical than traditional sensors, we designed a bending sensor based on seven-core six-mode fiber power monitoring. By splicing and tapering the seven-core six-mode fiber, the coupling degree and bending sensitivity of the fiber mode are significantly improved. Through simulations, we found that with the increase in the taper ratio, the mode coupling process between the cores is accelerated, thereby shortening the transmission distance required to reach the optimal “coupling point”. In addition, we studied the effect of bending on the power distribution of the seven-core six-mode fiber after tapering and determined that the fiber is most sensitive to bending when the taper ratio is 2.8 and the taper length is 4.84 mm. The distribution of the core power in the seven-core six-mode fiber with different bending radii is analyzed according to the simulation data. We found that the sensor can achieve high-sensitivity bending sensing within the bending radius range of 0.1 m to 0.4 m (curvature range of 2.5 m^−1^ to 10 m^−1^). Based on the central symmetric structure of the seven-core six-mode fiber, the change in each side core power can characterize the bending direction of the fiber. We designed a deep regression network, BendNet, and used machine learning to predict the bending shape of the optical fiber through the power variation in each core. The results show that the regression model has good prediction and explanation capabilities for optical fiber bending. The seven-core six-mode optical fiber bending sensor has a simple structure and is easy to design. It realizes accurate vector sensing under a large bending radius and effectively solves the problem of small curvature sensing of traditional sensors. Compared with traditional sensors, the design based on power analysis simplifies the sensing method, improves the response speed, and expands the scope of application.

## Figures and Tables

**Table 1:** Sensitivity of power ratios in different regions to curvature.

Curvature (m^−1^)	1~2.5	2.5~10	10~20
Core No. 3 (/m^−1^)	0.039	0.163	0.021
Core No. 2/4 (/m^−1^)	0.044	0.150	0.027
Core No. 5/7 (/m^−1^)	0.041	0.140	0.034
Core No. 6 (/m^−1^)	0.054	0.166	0.012

**Table 2:** Performance comparison of current mainstream sensors.

Sensor Type	Curvature Range (m^−1^)	Sensitivity	Vector Capability	Structural Complexity	System Cost	Reference
FBG (Schulze, 2018)	0–5	/	Scalar	Low	Low	[12]
FBG (Choi, 2025)	0–5	~0.1 nm/m^−1^	Scalar	High	Medium	[14]
LPG (Bhatia, 1999)	0–6	~0.1–0.25 nm/m^−1^	Scalar	Low	Low	[15]
MZI (Li, 2012)	0–5	~0.05–0.1 nm/m^−1^	2D Vector	Medium	Medium	[17]
Directional Torsion Sensor (Song, 2023)	0–3	~0.15 nm/m^−1^	2D Vector	High	Medium	[19]
Proposed 7-Core Taper Sensor	2.5–10	≥0.14/m^−1^	2D Vector	Low	Medium	/

## Data Availability

The data presented in this study are openly available in GitHub at https://github.com/HUST-IOF/Bendnet, accessed on 17 December 2025.
